# Incomplete Kawasaki Disease in an Adult Complicated by Stroke

**DOI:** 10.7759/cureus.72469

**Published:** 2024-10-27

**Authors:** Asif Dabeer Jafri, Chitralekha Naik, Srikant K Dhar, Mareya Zeenat, Kaneez Fatima

**Affiliations:** 1 Internal Medicine, SUM Ultimate Medicare, Bhubaneswar, IND; 2 Neurology, SUM Ultimate Medicare, Bhubaneswar, IND; 3 Internal Medicine, Institute of Medical Sciences and SUM Hospital, Bhubaneswar, IND; 4 General Practice, SUM Ultimate Medicare, Bhubaneswar, IND; 5 Radiology, All India Institute of Medical Sciences, Bhubaneswar, Bhubaneswar, IND

**Keywords:** coronary aneurysm, incomplete kawasaki disease, intravenous immunoglobulin (ivig), ischemic stroke, nonpurulent conjunctivitis

## Abstract

Kawasaki disease (KD) is a systemic vasculitis with an unclear origin primarily affecting young children. The sole difference between complete and incomplete KD upon presentation is the number of signs and symptoms. There is a concerning case of a 50-year-old male with incomplete KD who has additionally presented with a stroke. Cases of KD are uncommon in adults but well-known in children. Ischemic stroke is quite rare in people with incomplete KD. This appears to be the first documented case of incomplete KD linked to a stroke in an adult. Because of the potentially life-threatening consequences associated with KD, healthcare professionals must take a multidisciplinary approach when assessing and managing such patients.

## Introduction

Kawasaki disease (KD) was named in honor of Tomisaku Kawasaki, a Japanese pediatrician who initially identified this febrile vasculitis in 1967. It is an acute multi-systemic vasculitis involving medium- to small-size arteries presenting as a self-limited febrile illness of unknown cause. Children below five years are the most common group affected. In developed nations, it is replacing rheumatic heart disease as the most common cause of acquired heart disease. To reach a diagnosis, we inherently rely on diagnostic criteria, which encompass the usual combination of signs and symptoms defined by the Japanese Kawasaki Research Committee or the American Heart Association [1,2]. There is no specific diagnostic test available for KD; the presence of a fever lasting five days or more and at least four of the following five clinical symptoms are traditionally used to diagnose KD. These are 1) changes to the eyes, including bilateral non-exudative painless conjunctivitis; 2) changes to the mouth and lips, including strawberry tongue and erythematous cracked lips; 3) a polymorphic rash covering the perineum, trunk, and extremities; 4) alterations to the extremities, such as erythema in the hands and feet in the first week and desquamation in the hands and feet the next two to three weeks; and 5) unilateral cervical lymphadenopathy with a diameter larger than 1.5 cm. Five or more days of fever with at least two of the clinical criteria discussed above for classical KD are considered to be the criteria for incomplete or atypical KD [3]. This term should also be used to describe cases that have symptoms that are uncommon in classical KD, such as the acute surgical abdomen, renal impairment, and pleural effusion. Some supplemented laboratory criteria increase the probability of KD if present. These included raised liver enzymes, anemia, elevated inflammatory markers, thrombocytosis, leukocytosis, hyponatremia, sterile pyuria, hypoalbuminemia, and cerebrospinal fluid (CSF) pleocytosis.

## Case presentation

A 50-year-old male presented to the emergency department with the chief complaint of persistent high-grade fever for four days, which was associated with a one-day history of sudden onset of weakness and decreased sensation on the right side of the body involving both upper and lower limbs. He had no past medical history of any chronic illness and had not traveled or received any vaccinations recently. Before he visited our hospital, he had received a course of antibiotics for his symptoms, but there was no improvement, and his condition remained unchanged. On general examination, he was conscious, oriented, and febrile with a temperature of 102°F. There was a bilateral, non-exudative, painless bulbar conjunctival injection. On inspection of his mouth, there were ulcers present over the oral cavity and pharynx, which were painful; the tongue was swollen and erythematous like a strawberry tongue; and there were erythematous rashes present over the trunk and limbs. He had no cervical lymphadenopathy. Neurological examination revealed right-sided weakness (grade 4/5) with decreased sensation to touch, pain, and temperature without any bowel and bladder involvement; examination of all cranial nerves was normal. Additionally, he had experienced similar symptoms (fever, conjunctival chemosis, and oral ulcers) 15 days prior, leading to his admission to a hospital. Investigations conducted to identify the source of the infection yielded no conclusive results. He was treated at that facility as a case of pyrexia of unknown origin and received a regimen of antibiotics, antivirals, and antifungals. His symptoms continued unabated despite receiving treatment. As a result, he was referred to our hospital for further assessment and management. Upon reviewing his medical history and conducting a physical examination, we considered a broad spectrum of differential diagnoses, primarily encompassing infectious, inflammatory, and autoimmune conditions; however, none could be definitively confirmed. After a thorough multidisciplinary discussion involving immunologists, neurologists, and infectious disease specialists, we concluded that, despite being uncommon in adults, KD was the only condition that could fully explain all of his symptoms. This led to the diagnosis of incomplete KD.

**Figure 1:**
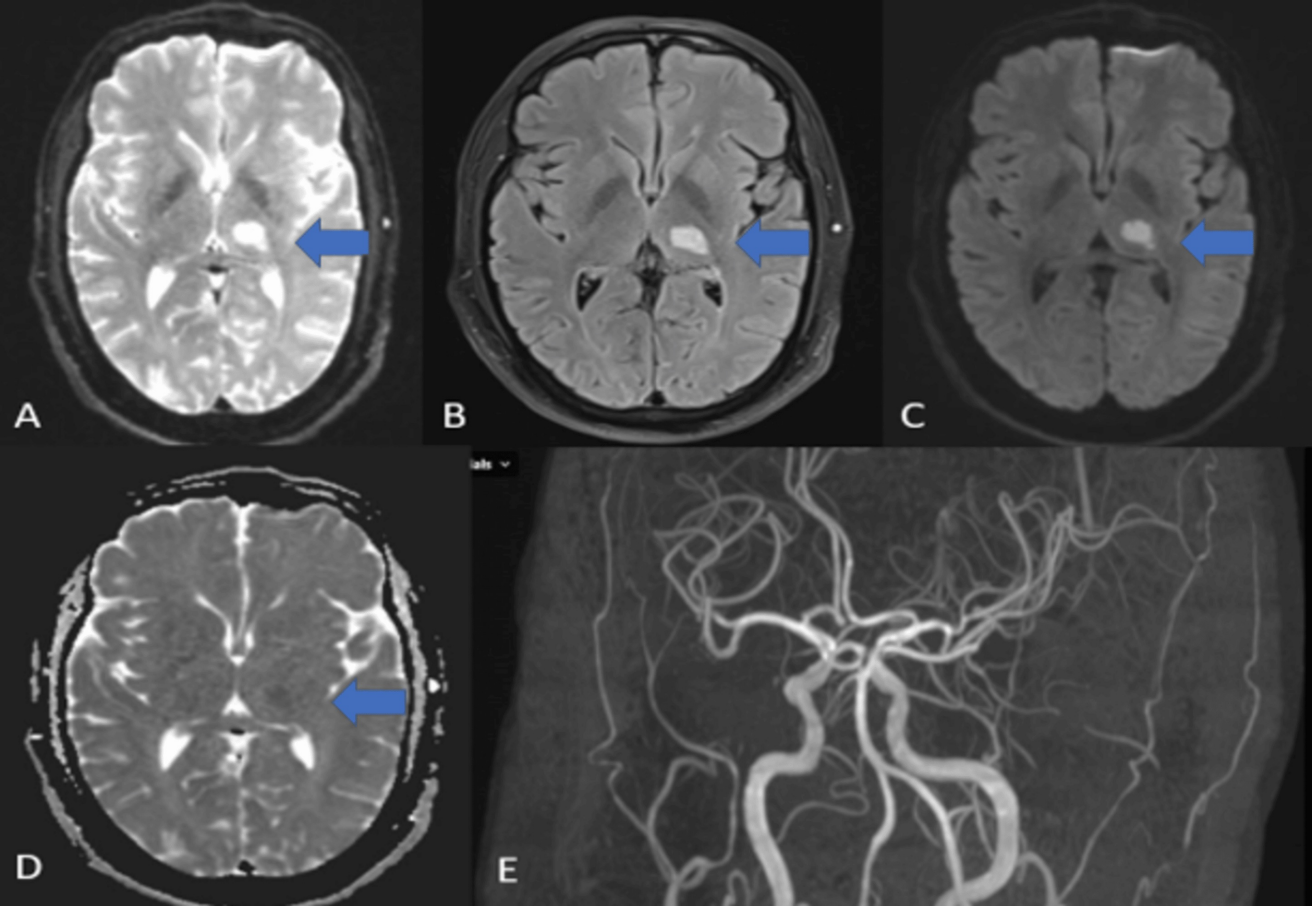
(A) T2 weighted imaging; (B) T2 FLAIR MR imaging shows a hyperintense lesion (blue arrow) in the left thalamus; (C) DWI shows an increased signal in the left thalamus (blue arrow); (D) Reversal on the corresponding ADC map as shown by (blue arrow); (E) 3D TOF MR angiogram revealed normal cerebral vasculature FLAIR: fluid-attenuated inversion recovery; DWI: diffusion-weighted imaging; ADC: apparent diffusion coefficient; TOF: time of flight

The laboratory parameters are displayed in Table 1. The kidney function test, viral hepatitis panel, and lipid profile were within the normal limit. Antibodies for HIV types 1 and 2 returned negative results. Testing for tropical diseases, including malaria, dengue, Leptospira, and scrub typhus, also yielded negative results. Tests for Epstein-Barr virus and cytomegalovirus were negative as well. A CSF analysis was performed, which indicated normal findings, and the CSF viral panel was negative. Cultures from blood, urine, and CSF were all sterile. Serum procalcitonin levels were within the normal range. Anti-nuclear antibodies (ANA) and the extractable nuclear antigen (ENA) panel results were normal. Vasculitis panels for c-antineutrophil cytoplasmic antibody (ANCA) and p-ANCA were also within normal limits. The COVID-19 reverse transcription-polymerase chain reaction (RT-PCR) test returned a negative result. The anti-streptolysin O (ASO) titer was negative, and anti-cyclic citrullinated peptide (anti-CCP) and rheumatoid factor antibodies were found to be within normal limits. Magnetic resonance imaging (MRI) of the brain identified a small acute infarct in the left thalamus, as depicted by the blue arrows, while the magnetic resonance angiogram (MRA) of the brain and neck vessels showed no significant abnormalities as seen in Figure 1. Due to the presence of an oral ulcer and abdominal pain, an upper gastrointestinal (UGI) endoscopy was conducted, which revealed an esophageal ulcer. High-resolution computed tomography (HRCT) of the chest demonstrated patchy linear fibrotic changes in the bilateral upper lobes, right middle lobe, and bilateral lower lobes, suggestive of sequelae from previous infection. No active ground glass opacities or consolidation in bilateral lungs. CT abdomen and echocardiography were normal.

**Table 1:** Laboratory parameters at admission S. Na: serum sodium; S. K: serum potassium; SGOT: serum glutamic oxaloacetic transaminase; SGPT: serum glutamate pyruvate transaminase; TSH: thyroid stimulating antibody

Analyte	Patient result	Reference value
Hemoglobin	11.9	13-17 g/dl
Total leucocyte count	9.42	4-11 x 10^9/L
Total platelets count	451	150-400 x 10^9/L
S. Na	137	135-145 mEq/ L
S. K	3.8	3.5-5 mEq/L
SGOT	123.5	5-40 U/L
SGPT	127.1	5-40 U/L
T. bilirubin	0.89	0.0- 2.0 mg/dl
Alkaline phosphatase	106	40-129 IU/L
T. protein	6.49	6.0-8.3 gm/dl
S. albumin	2.8	3.3-5.2 mg/dl
Urea	20	13-45 mg/dl
Creatinine	0.8	0.5-1.5 mg/dl
Calcium	8.4	8.6-10.3 mg/dl
TSH	1.90	0.270-4.20 mIU/L

**Figure 2:**
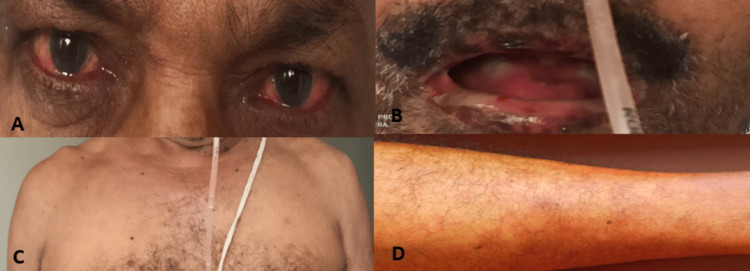
(A) Nonpurulent conjunctivitis; (B) Strawberry tongue with an ulcer over lips; (C, D) Polymorphic rashes over chest and leg

**Figure 3:**
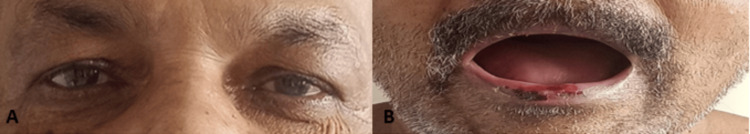
(A, B) Complete resolution of conjunctival injection and oral lesions after treatment

The patient received a high dosage of enteric-coated aspirin, specifically 325 mg every eight hours, in conjunction with intravenous immunoglobulin (IVIG) administered at a rate of 2 g/kg over five days. Remarkably, the patient was afebrile and exhibited improved health the day following the initiation of treatment, which is an impressive result for the combination of immunoglobulin and aspirin. Throughout his hospital stay, his symptoms gradually diminished, accompanied by a consistent enhancement in his overall condition and the normalization of inflammatory markers. There was no need for additional IVIG, corticosteroids, or immunosuppressive therapies, as the patient did not experience any persistent or relapsing symptoms. After receiving inpatient care for one week, he was discharged on low-dose aspirin and a proton pump inhibitor. Three weeks later, a computed tomography coronary angiography was done, which revealed no coronary aneurysms. The patient is currently undergoing regular follow-up and is in good health. Figures 2-3 illustrate the images taken before and after treatment.

## Discussion

KD is a febrile, inflammatory vasculopathy of unknown cause that targets small and medium-sized arteries of the body and can affect any organ. It is mainly of two types: typical, also known as classical KD, which is more common, and atypical, also known as incomplete KD, which is less common. In complete KD, there is a fever for at least five days and at least four of the five clinical criteria mentioned above. In incomplete KD, there is a fever for a five-day duration along with two or three of the major clinical criteria. Since the vasculitis in KD is systemic, it can involve multiple systems. A biopsy is seldom required and not essential for diagnosis. The most frequent organ involvement in KD is the heart, with coronary arteries being the most common vessel affected in the form of coronary aneurysm.

Neurological involvement in KD is poorly understood and has only been recorded in a few case studies. Involvement of the central nervous system takes place more often in children than in adults [4]. Stroke associated with KD is extremely rare and exceptional, with just a few reported cases of KD in children resulting in ischemic stroke [5-10]. Therefore, these reports support our case, as stroke can happen with KD. Nine patients who met the criteria for an incomplete disease were evaluated in a French study; most of the symptoms observed were similar to our case and included fever, changes in the extremities, skin rashes, changes in the oral cavity, and conjunctivitis [11,12].

In KD, coronary aneurysms were identified in merely 19% of instances, whereas coronary vasculitis was observed in 26% of the cases [13,14]. Thus, KD can occur without involving coronaries, as in our case. Preventing coronary artery aneurysm (CAA) formation and symptom relief are the main treatment approaches for KD. The mainstay of treatment is thought to be IVIG infusion and supportive inpatient care [15,16]. There are now several IVIG resistance risk scores that can be used to identify individuals who are at a high risk of not responding to IVIG treatment, which is strongly linked to the emergence of CAA. According to American Heart Association guidelines for IVIG-resistant cases, a second course of IVIG should be tried along with IV corticosteroids at the dose of 2 mg/kg, switching to an oral dose once the patient becomes afebrile. Oral steroids should be continued till CRP normalizes. Tumor necrosis factor (TNF)-alpha antagonists like infliximab can be used as second-line therapy. Other medications that are used variably include interleukin inhibitors (anakinra), plasma exchange, and cyclosporine. In severe refractory disease, cyclophosphamide should be tried as the last option [17].

Aspirin has been the treatment of choice due to its antiplatelet effects; in patients with small CAAs, a high-dose regimen for an initial period is followed by a decreased dose for an extended period, while dipyridamole has been suggested for those with larger CAAs [18]. The AHA recommends that these individuals take low-dose aspirin therapy until it is established that aneurysms have disappeared. Falcini et al. [19] conducted a study and found that adults with a history of KD without CAA had excellent long-term outcomes. This situation is comparable to ours because of his successful recovery from the stroke.

In the absence of CAA, several studies advise stopping all drugs right away after the acute period [20]. However, our patient continued to take antiplatelets due to stroke. The diagnostic difficulties of incomplete KD arise from patients not fulfilling all the established criteria, which could result in treatment delay and increased chances of complications like the stroke this patient experienced. Besides fever, our patient only had three of the four required symptoms (conjunctivitis, mucositis, and skin rashes); thus, he did not meet the classic KD criteria. On the other hand, leukocytosis, thrombocytosis, and slightly decreased serum albumin levels were also indicative of incomplete KD.

Typically, not all of the clinical symptoms occur at once, and it can be difficult to make a diagnosis relatively early in the course of the illness. As far as our knowledge is concerned, there has not been a single case of incomplete KD in an adult complicated by stroke. Hence, the majority of adult KD patients in many developing nations continue to go untreated, which is probably because healthcare professionals are unfamiliar with this rare condition.

## Conclusions

Incomplete KD is a treatable condition that can be prevented from developing potentially life-threatening complications like stroke with early diagnosis and a proper multidisciplinary approach. In complicated cases that appear as autoimmune, inflammatory, or infectious disorders, adult-onset KD should be taken into consideration as a differential diagnosis. Our case highlights the significance of considering KD at any age. This case also highlights the significance of upholding a heightened sense of suspicion for KD in patients who present with prolonged fever and suggestive clinical features, even if the entire criteria are not satisfied. Early detection and effective treatment of this potentially lethal condition are crucial for preventing KD-related complications.
